# Integrating genome size variation, ISSR-derived genetic structure, and phenotypical traits from the Southern Peruvian Andean maize (*Zea mays* L.) race *Cabanita*

**DOI:** 10.3389/fpls.2026.1798629

**Published:** 2026-06-26

**Authors:** Miguel Vera-Vega, Gastón Zolla, Lena Gálvez Ranilla

**Affiliations:** 1 Grupo de Investigación en Fisiología Molecular de Plantas del PIPS de Cereales y Granos Nativos, Facultad de Agronomía, Universidad Nacional Agraria La Molina, Lima, Peru; 2 Laboratory of Research in Food Science, Universidad Catolica de Santa Maria, Arequipa, Peru

**Keywords:** *Cabanita* race, genome Size, ISSR marker, Peruvian Andean maize, phenotypical traits, *Zea mays* L

## Abstract

**Introduction:**

*Cabanita* maize is a native race with relevance for food security and economic subsistence among Southern Andean communities in Arequipa (Peru). Generally, studies are targeted to evaluate the genetic diversity of several maize races in Peru and elsewhere; however, the genetic diversity within a single maize race has not been explored yet.

**Methods:**

This study evaluated for the first time the genetic and genome size (2C DNA) variability of 48 *Cabanita* maize accessions from two provinces (Castilla and Caylloma) of the Andean region of Arequipa (Peru), using 7 ISSR markers and cytometric methods. In addition, preliminary marker-phenotypical trait relationships were explored.

**Results and Discussion:**

Despite the open-pollinated traditional cultivation of this crop, *Cabanita* accessions showed low genetic variability (PIC values 0.130-0.140) and low variation of their 2C DNA contents (5.35-5.65 pg). However, intra-racial genetic differences were revealed in the ISSR-based dendrogram and showed relation with the province of origin. UBC812, A5, and UBC840* markers were the most informative for evaluating genetic diversity based on their genetic diversity parameters. Castilla accessions showed slightly higher genetic diversity and 2C DNA contents and coefficients of variation (CV) than Caylloma samples. Preliminary analysis of marker-phenotypical trait relations revealed that UBC810 and UBC840* markers had relation with some *Cabanita* maize traits at phenotypical (kernel weight, ear length and number of rows) and chemical (free and dietary fiber-linked hydroxycinnamic acid derivative compounds) levels. However, this needs further validation through future genome-wide association studies (GWAS) to better characterize this underrepresented Andean maize race. This information would be relevant for future breeding programs at local level and product development to enable the potential designation of origin, enhancing the livelihoods of Southern Andean farmers in Peru.

## 1 Introduction

The Andean region is a worldwide hotspot of biodiversity including a wide array of crop species such as maize (*Zea mays* L.) (Myers et al., 2000). Peru is a key secondary center of maize diversification worldwide (Gamarra et al., 2011). The genetic diversity of Peruvian maize is divided into 52 races based on new collections made from 2013 to 2016 (Ministry of Environment, 2018). Almost 44% of the total race diversity in Peru is adapted to Andean ecosystems (Ministry of Environment, 2018). In fact, the Andean region has the highest maize race phenotypical diversity in terms of variable ear and kernel morphology and pigmentations (Ministry of Environment, 2018). Most Peruvian Andean communities rely their economic subsistence on maize cultivation, and main forms of consumption and commercialization are in fresh-immature or dried-mature forms (INEI, 2024). It has been highlighted that the preservation and characterization of Latin American native maize diversity is an important strategy for guaranteeing worldwide food security and climate change resilience (Guzzon et al., 2021). Thus, the scientific research about these strategic resources is fundamental.

Studies related with the characterization of Peruvian maize diversity are scarce and overall targeted a fraction of existing races. Plant and internal ear traits have been used to classify some Peruvian maize races (Ortiz et al., 2008a, b). More recently, the population structure based on genotyping-by-sequencing and single-nucleotide polymorphisms (SNP) markers has been investigated in accessions corresponding to 5–9 races, revealing high genetic diversity among evaluated Peruvian races (García-Mendoza et al., 2025; Arbizu et al., 2025). However, intra-racial phenotypical differences have been also observed in maize from Peru and other Andean countries (Ministry of Environment, 2018; Tapia et al., 2021). A maize race has a specific geographical and ecological adaptation (Wellhausen et al., 1952), along with identified cultural uses (Ministry of Environment, 2018). The conservation of a maize race by Andean communities is deeply correlated with its economic value. The molecular basis about the phenotypical differences within a Peruvian maize race has not been well explored. Therefore, the scientific information about a single maize race would be of critical relevance for specific Andean regions. This could be the base of future initiatives aimed at improving the *in-situ* and *ex-situ* preservation and at developing local breeding strategies for the benefit of Andean communities.

*Cabanita* is the name of an Andean maize race that has been traditionally cultivated by Southern Andean indigenous communities located in the region of Arequipa (Peru) at altitudes around 3000 m, and where it is considered a staple crop (Fuentes-Cardenas et al., 2022). This maize race was not included within the first classification of the Peruvian maize genetic diversity reported by Grobman et al. (1961). This may have led to the low interest in its preservation and research. Later, the Peruvian Ministry of Environment included this specific race in the second Peruvian maize race classification likely due to its high cultural relevance for Southern Andean communities (Ministry of Environment, 2018). It has been proposed that the cultivation of *Cabanita* maize extends not only in the Arequipa region but also to other regions of Southern Peru such as Moquegua and Tacna (Ministry of Environment, 2018). However, this distribution has not been confirmed since the scientific information about this maize race is very limited. The first initiative for the study and preservation of *Cabanita* maize started in 2019, when 48 accessions were collected from the Arequipa region (Fuentes-Cardenas et al., 2022). This material is currently preserved at Universidad Catolica de Santa Maria (Arequipa). *Cabanita* maize has been reported as a potential source of phenolic and carotenoid bioactive compounds (Fuentes-Cardenas et al., 2022). In addition, phenotypical differences mainly related to their variable kernel morphologies and pigmentations including red, orange and white phenotypes have been also highlighted (Ranilla et al., 2023). Nevertheless, the genetic diversity of *Cabanita* maize race and its relation with found phenotypical differences have not been investigated yet. Current genetic improvement programs in Peru are mainly focused on the improvement of *Kculli* (purple maize) and *Cusco Gigante* races due to their commercial importance (Medina-Hoyos et al., 2020; Soto-Aquino et al., 2024; Ministry of Environment, 2018). No research has been carried out in case of *Cabanita* maize at genetic level; therefore, no breeding programs have been developed using this crop. Future research engaging Andean people for ensuring the *in situ* preservation of *Cabanita* maize along with participatory breeding strategies may play a role in the socio-economic development of these communities (Ceccarelli, 2015).

DNA markers such as Inter-Simple Sequence Repeat (ISSR) markers have been applied to determine the genetic diversity of plant germplasm from different origins worldwide (Alves et al., 2025; Esquivel-Chi et al., 2026; Gao et al., 2025). These markers are polymorphic, cost-effective, simple to implement, they can detect DNA variation regardless of sequence data and may be useful in underfunded regional conservation programs (Azizi et al., 2021). ISSR markers have been used for the determination of the genetic diversity in Saudi Arabian, Mexican, and Latin American maize populations (Amal et al., 2025; Hernández et al., 2025; Do Amaral Júnior et al., 2011).

In addition to the sequence-level diversity, the nuclear DNA content also reflects plant evolution and adaptation. The genome size (2C DNA), defined as *the total content of DNA in an unreplicated gametic nucleus*, has shown variation in plant species depending on different ecological, physiological and evolutionary factors (Faizullah et al., 2021; Pellicer et al., 2018). In case of maize, the determination of 2C DNA has helped to understand the role of DNA repair mechanisms during kernel development (Pedroza-Garcia et al., 2021), to reveal structural chromosome alterations (Silva et al., 2020), or to evaluate its correlation with phenotypical and environmental factors (Jian et al., 2017; González and Poggio, 2021). The 2C DNA of Peruvian maize races along with its association with genetic, metabolic, phenotypical, and ecological diversity remains unknown.

To advance with the research about the Andean maize race *Cabanita*, the objectives of this next stage study are a) to determine for the first time its genetic diversity based on ISSR molecular markers, b) to evaluate its genome size variability, and c) to analyze at preliminary level the potential relations between molecular data with kernel, ear, and metabolite phenotypical traits previously evaluated in 48 accessions of *Cabanita* maize (Fuentes-Cardenas et al., 2022).

## 2 Materials and methods

### 2.1 Plant material

This study evaluated 48 accessions of *Cabanita* maize previously collected from two provinces (Caylloma and Castilla) located in the Southern Andean region of Arequipa (Peru) (Fuentes-Cardenas et al., 2022). This material is currently under refrigerated storage (5 °C) at Universidad Catolica de Santa Maria (Arequipa, Peru). Information about the collection procedure, the geographical coordinates, along with the environmental conditions of each locality are found in Fuentes-Cardenas et al. (2022). **Supplementary Table 1** shows the new accession codes used in current study, their previous equivalent codes, the localities of origin per province and districts of all material. **Supplementary Table 2** exhibits the average values of environmental conditions (maximum and minimum temperatures, rainfall, and relative humidity) during the period of cultivation of *Cabanita* maize plants based on the study of Fuentes-Cardenas et al. (2022). New pictures of each collected accession with details of kernel pigmentation differences are shown in **Supplementary Presentations 1** and **2**.

### 2.2 Phenotypical trait data of *Cabanita* maize accessions

The phenotypical data corresponding to kernel and ear physical characteristics and the phenolic and carotenoid profiles from each evaluated accession were previously obtained (Fuentes-Cardenas et al., 2022). In that study, only the average values of evaluated physical and metabolomic profiles per locality and maize type were reported. In current work, the whole data from the 48 accessions were used, including new information about the kernel color as will be described in next item. The complete data file is shown in **Supplementary Data 1**.

### 2.3 Kernel color analysis

The color parameters (L*, a*, b*) based on the CIELAB scale of kernels from the 48 maize accessions (five measurements per accession) were analyzed with a spectrophotometer CM-5 (Konica-Minolta, Japan). The D-65 light source and the observer angle of 10° were used according to Salinas-Moreno et al. (2021).

### 2.4 DNA extraction and ISSR genotyping

#### 2.4.1 DNA isolation

Kernels (10 units) from each of the 48 accessions were sown at a depth of 1.5 cm in seedling trays containing PLUGMIX substrate (KLASSMAN), primarily composed of oligotrophic Sphagnum peat moss with silt, NPK mineral fertilizers and trace micronutrients. The substrate’s pH was 6.0 ± 0.3, and electrical conductivity was 0.3–0.4 mS/cm, as specified by the manufacturer. The trays were watered with deionized water (pH: 7.43; EC: 1.502 µS m^-1^), then placed in the growth chamber, where growing conditions were set at 22 °C, 16 h light/8 h dark photoperiod, light intensity of 150 µmol m^-2^ s^-1^ from LED lamps, and 55% relative humidity. The leaf tissue per accession was collected at 2 weeks of age and dried on silica gel for one week. The dried tissue was ground using a bead mill to a fine powder and then transferred to a 1.7 mL vial. For cell lysate preparation, 700 μL of CTAB extraction buffer was added along with β-mercaptoethanol (700:5), and the mixture was incubated at 65 °C for 45 min. Next, this lysate was mixed with chloroform:isoamyl alcohol (24:1) and centrifuged at 7000 rpm for 5 min to separate the DNA into the aqueous phase. An aliquot of 750 μL of this phase was then transferred to a new tube containing 700 μL of absolute ethanol. The samples were incubated at -20 °C for 30 min and then centrifuged at 7000 rpm for 20 min to precipitate the DNA. The precipitated DNA was washed twice with 90% ethanol, centrifuged at 7000 rpm for 5 min after each wash, and allowed to dry at room temperature for 2 h. The pellet was dissolved in 50 μL of ultrapure water at 65 °C for 10 min. Finally, 5 μL of RNase were added to the samples, and then were incubated at 37 °C for 30 min before further analysis.

#### 2.4.2 ISSR amplification

Seven ISSR primers A5, UBC817, UBC840, UBC840* (Valiyeva et al., 2019), P-ISSR14 (Barcaccia et al., 2003), UBC810, and UBC812 (Muhammad et al., 2017) were used to evaluate the genetic diversity of the 48 *Cabanita* accessions. These primers were selected based on their high rate of polymorphism shown in other studies performed in maize (Valiyeva et al., 2019; Barcaccia et al., 2003; Muhammad et al., 2017). Selected ISSR primers showed clear and polymorphic patterns following the experimental protocol. The primer sequences are shown in **Supplementary Table 3**. Amplification reactions were performed using GoTaq^®^ Green Master Mix (Promega). The PCR protocol included an initial denaturation at 95 °C for 2 min, followed by 34 cycles at 94 °C for 1 min, an annealing temperature specific to each primer (**Supplementary Table 3**) for 1 min, and an extension at 72 °C for 2 min. The process was completed with a final extension at 72 °C for 7 min.

#### 2.4.3 Gel electrophoresis and scoring of bands

Agarose gels (3%) were prepared in 1X TBE buffer and used to separate the amplicons. Electrophoresis was performed at 8 V for 4.5 h. The bands were observed under UV light and documented to generate the binary matrix by identifying presence (1) and absence (0), discarding ambiguous and weak bands. To guarantee the reproducibility and robustness of the markers, only bands within the region between 300 and 1100 pb were considered to exclude low molecular weight PCR artifacts and low intensity bands in larger fragments, which are generally characterized by being erratic (Bornet and Branchard, 2001). The resulting matrix was used for the analysis of diversity, distance, and population genetic structure, including the construction of the dendrogram (This matrix is shown in **Supplementary Data 2**).

### 2.5 Genetic diversity and structure analysis

The genetic diversity parameters were calculated from the binary matrix generated by the seven ISSR primer pairs under study. The number of alleles per locus (Na), effective number of alleles (Ne), expected heterozygosity (He), polymorphic information content (PIC), and Shannon index (I) were estimated for each ISSR primer and for each province (Caylloma and Castilla). The structure of genetic variation was assessed using an Analysis of Molecular Variance (AMOVA). Furthermore, to infer genetic similarity among the 48 accessions, a dendrogram was constructed based on the matrix derived from the Spearman distance and the UPGMA clustering method (Stavridou et al., 2021). The Spearman distance is derived from the Spearman rank correlation coefficient, which is a measure of the monotonic relationship between two variables. This parameter showed better biological consistency when compared with the other standard genetic distance options (that were also tested) (Bouland et al., 2023; Crispino et al., 2023; Jaskowiak et al., 2013). This approach revealed a clear discrimination of *Cabanita* maize accessions based on the province of origin. All analyses were performed using InfoGene software (https://www.info-gen.com.ar/).

### 2.6 Genome size estimation (2C DNA)

The 2C DNA content was measured in four individuals from each accession. *Pisum sativum* cv. citrad (9.09 pg) was used as an internal standard, kindly provided by Dr. J. Doležel from the Institute of Experimental Botany, Sokolovská, Czech Republic (Doležel et al., 2007). For each individual, 1 cm^2^ from a young leaf maize samples were co-chopped with the same amount of tissue of *P. sativum* leaves in a Petri dish with 1 mL of buffer Otto I (citric acid 0.1 M and 0.5% v/v of Tween 20), using a stainless-steel razor blade until obtaining a homogeneous sample. The solution obtained was filtered through a 40 µm nylon mesh and centrifuged at 1500 rpm, 5 °C for 5 min. The supernatant was removed and the pellet was resuspended in 500 µL of Otto I and 500 µL of Otto II (400 mM Na_2_PO_4_.12H_2_O, pH 8–9) was added, supplemented with 50 µg/mL of propidium iodide (Sigma-Aldrich) and 50 µg/mL RNase (Sigma-Aldrich). The nuclear suspension was incubated at 5 °C for 10 min and analyzed in an Attune Nxt flow cytometer (Thermo Fisher Scientific, MA, USA) where at least 5,000 nuclei were counted in each run. Three readings were made for each sample, and the content of nuclear DNA was calculated as follows: Sample value 2C (DNA pg) = reference value 2C x (position of the mean peak of sample 2C/position of the mean peak of reference 2C). A coefficient of variation (CV) below 5% was considered indicative of acceptable instrument performance (**Supplementary Data 3**). This service was provided by Laboratorio de Biotecnología y Mutación del PIPS en Cereales y Granos Nativos –UNALM. Finally, Spearman correlation analyses were conducted in R (version 4.0.2) to examine the associations between GS and geographic factors (latitude, longitude, and altitude) and other phenotypical traits.

### 2.7 ISSR–phenotype relations

Phenotypical trait range values of accessions belonging to each identified genetic group (12) were compared, and differences among groups were identified. Markers with consistent contrasting bands were selected. The potential associations between ISSR markers and phenotypical traits were examined using a one-way ANOVA (Al-Daej et al., 2023), considering each band as a dominant marker coded as present (1) or absent (0). These markers were treated as independent factors (treatments). For each trait, differences in phenotypical means were evaluated between the groups defined by each marker. The effect of the marker was considered significant when p<0.01 (Habib et al., 2024). To strengthen the identification of markers related to the phenotypical traits under study, the False Discovery Rate (FDR) was used. This parameter allows an adjustment for multiplicity, guaranteeing the reproducibility of trait-genotype associations and prioritizing true discoveries, even in the face of the complexity of the polygenic architecture of the analyzed characters (Sesia et al., 2021). The selection of significant preliminary marker-phenotypical trait relations within the set of evaluated 48 *Cabanita* accessions were carried out considering both p<0.01 and FDR<0.05 (Yang et al., 2005). All analyses were performed using Infostat software (https://www.infostat.com.ar/).

## 3 Results

### 3.1 Genetic diversity and structure using ISSR primers

**Figure 1:**
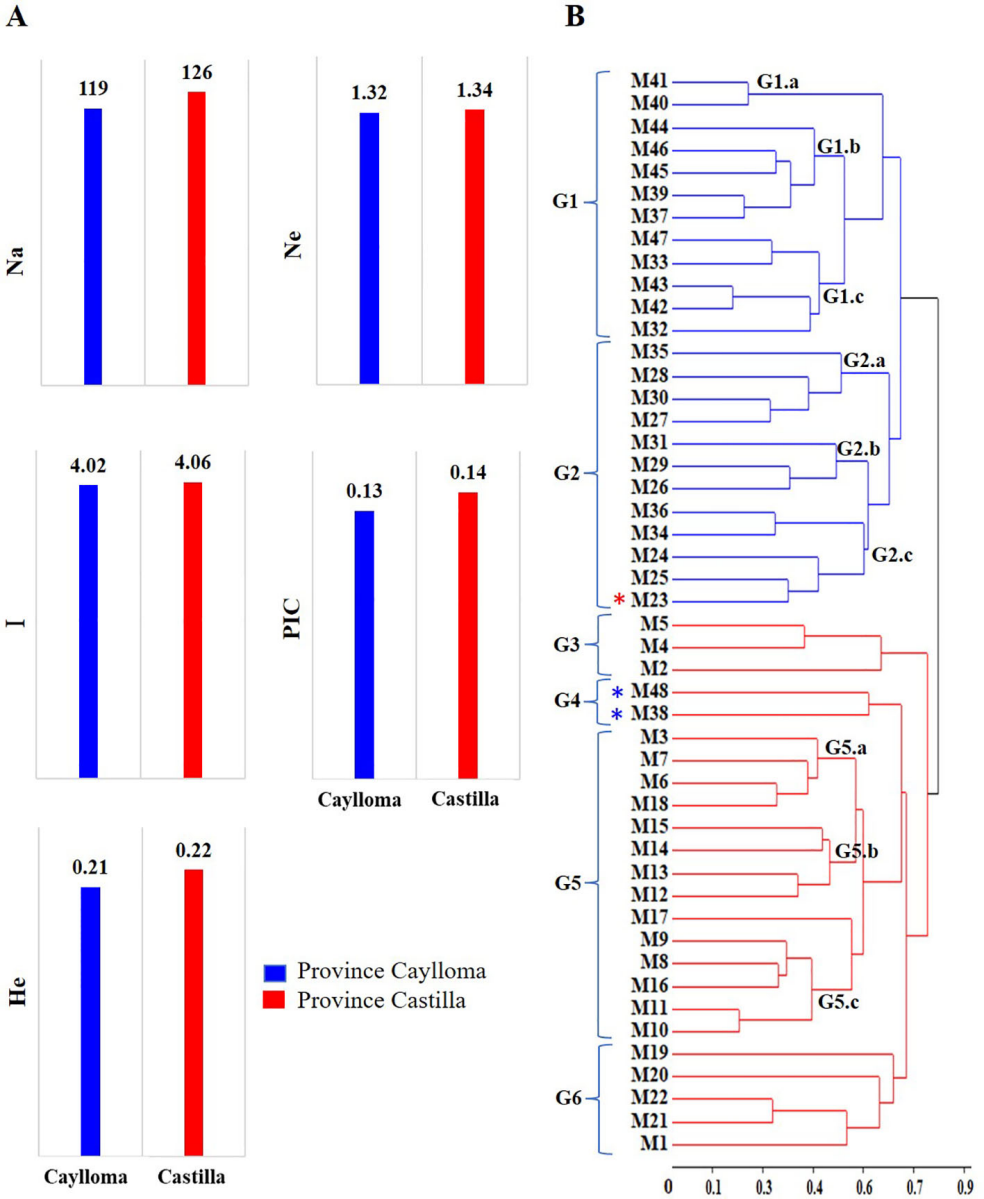
Genetic diversity parameters and genetic structure of 48 accessions from the Peruvian Andean maize race *Cabanita*. **(A)** Na, number of alleles per locus; Ne, effective number of alleles; He, expected heterozygosity; PIC, polymorphic information content; I, Shannon Index. **(B)** Dendrogram generated based on 7 ISSR primers. Bars and genetic subclusters in red and blue correspond to maize accessions from Castilla and Caylloma provinces, respectively.

The genetic diversity indices associated with the ISSR primers (marker-based) used in the study are shown in **Table 1**. The indices (Na, Ne, He, I, and PIC) demonstrated that used markers had high discriminatory power. This enabled a robust molecular variability characterization of studied populations from *Cabanita* maize and the identification of intrapopulation variation (within provinces). **Figure 1** exhibits the dendrogram derived from 7 ISSR primers and the genetic diversity parameters related to the genotype found in the 48 *Cabanita* maize accessions. The genetic pattern shown in the dendrogram revealed a structure segregation based on the province of origin. Maize accessions were clustered in 2 large groups that corresponded to the provinces of Caylloma (blue group) and Castilla (red group). In addition, each province group presented 6 defined subclusters (12 groups in total). Only 3 *Cabanita* accessions (marked with an asterisk) (M23-Castilla, M38-Caylloma, M48-Caylloma), were clustered in opposite provinces of origin within the dendrogram. The genetic diversity parameters such as Na, Ne, and He were slightly higher in maize accessions from Castilla than those from Caylloma (**Figure 1**). In fact, from the six genetic subgroups showed in the dendrogram for maize from the Caylloma province, accessions from four subgroups corresponded to the same geographical locality (G1.a – Auqui, G2.a, G2.b, G2.c – mainly from Cusqui) (**Supplementary Data 1**). In the case of maize from the Castilla province, accessions from four genetic subgroups (from a total of six) (G5.a-Alleachaya-Pullugaya-Subna, G5.b-Ajocha-Subna, G5.c-Subna-Ajocha-Alleachaya, and G6-Huancarani-Alleachaya-Pulluguaya) derived from different localities (**Supplementary Data 1**). Despite the observed intra-racial genetic differences, PIC values of maize groups from Caylloma and Castilla provinces ranged from 0.13 to 0.14, respectively. The AMOVA (**Table 2**) confirmed the higher genetic variability within provinces (83.45%) than between them (16.45%).

**Table 1:** Genetic diversity indices associated with the 7 ISSR primers used for the study of accessions from the Peruvian Andean maize race *Cabanita*.

Primers	Na	Ne	I	PIC	He
A5	18 ± 0.00	1.46 ± 0.38	3.83 ± 0.99	0.78 ± 0.13	0.28 ± 0.17
P-ISSR14	17 ± 0.48	1.35 ± 0.50	3.85 ± 1.29	0.50 ± 0.15	0.20 ± 0.22
UBC810	23 ± 0.28	1.29 ± 0.41	3.85 ± 1.25	0.66 ± 0.11	0.19 ± 0.15
UBC812	21 ± 0.30	1.49 ± 0.37	3.84 ± 0.27	0.72 ± 0.14	0.29 ± 0.18
UBC817	14 ± 0.46	1.29 ± 0.49	3.85 ± 0.97	0.50 ± 0.14	0.18 ± 0.19
UBC840	16 ± 0.44	1.31 ± 0.38	3.85 ± 1.15	0.66 ± 0.13	0.21 ± 0.17
UBC840*	22 ± 0.00	1.14 ± 0.32	3.83 ± 0.66	0.91 ± 0.08	0.27 ± 0.12

Na, number of alleles per locus; Ne, effective number of alleles; I, Shannon index; PIC, polymorphic information content; He, expected heterozygosity.

**Table 2:** Analysis of molecular variance (AMOVA) based on the 7 ISSR primers used for the study of accessions from the Peruvian Andean maize race *Cabanita*.

Source of variation	Df^a^	Sum of squares	Estimated variability	Percentage of variation^*^
Among Province	1	86.32	2.97	16.45
Within Province	46	694.48	15.10	83.45
Total	47		18.07	100

a Df, degrees of freedom. ^*^P<0.0001.

### 3.2 Genome size (2C DNA)

The 2C DNA content in *Cabanita* maize accessions ranged from 5.35 to 5.65 pg, with the narrowest range (5.35 - 5.41 pg) observed in the province of Caylloma (**Table 3**). In addition, the variation of 2C DNA in relation to the obtained ISSR genetic structure is shown in **Table 4**. According to the coefficient of variation (CV) of the genome size, genetic groups were classified into three levels of variability: low (0.39% - 0.82%), medium (1.25% - 1.94%), and high (2.65% - 4.20%). Then, three nucleotypes were defined, and the groups G5.c and G5.a exhibited the highest CV values. The genetic groups corresponding to the province of Caylloma had CV ranging from low (G1.a, G1.b, G2.a, and G2.b) to medium (G1.c and G2.c). In contrast, those groups from Castilla showed overall CV values ranging from medium (G3, G4, G5.1b, and G6) to high (G5.a and G5.c). Additionally, **Table 5** shows that genome size variation correlated with some chemical (bound p-coumaric acid and free ferulic acid derivatives), phenotypical (ear center diameter, number of kernels per row, ear weight, and kernel color), and geographical (latitude and longitude) traits in *Cabanita* maize.

**Table 3:** Genome size (2C DNA) of accessions from the Peruvian Andean maize race *Cabanita* in function of their geographical origin (provinces, districts and locations).

Province	2C DNA (pg)	CV^a^ (%)	District^b^	Location^b^	Latitude^b^	Longitude^b^	Altitude^b^		2C DNA (pg)	CV (%)	
Castilla	5.45 ± 0.13	2.36	Andahua	Ajocha	S 15°29’57.2”	W 72°20’45.8”	3399	5.52 ± 0.04			0.78
				Huancarani	S 15°29’50.7”	W 72°20’48.6”	3347	5.42 ± 0.06			1.02
			Ayo	Subna	S 15°33’27.5”	W 72°14’16.3”	2845	5.39 ± 0.08			1.55
			Chachas	Alleachaya	S 15°30’04.1”	W 72°16’10.7”	3070	5.65 ± 0.11			2.03
				Pulluguaya	S 15°30’05.1”	W 72°16’14.4”	3043	5.35 ± 0.05			0.91
Caylloma	5.37 ± 0.07	1.37	Cabanaconde	Cusqui	S 15°37’30.9”	W 72°00’04.1”	2964	5.35 ± 0.05			0.88
				Liguay	S 15°38’01.6”	W 71°58’49.0”	3266	5.34 ± 0.05			0.98
				Auqui	S 15°37’05.6”	W 72°00’37.8”	3110	5.40 ± 0.11			1.99
				Ocollina - Tuntuiguita	S 15°37’41.6”	W 71°58’49.5”	3310	5.41 ± 0.08			1.47
				Huancce - Tranca	S 15°36’56.4”	W 71°58’19.5”	3332	5.31 ± 0.03			0.50

a CV: coefficient of variation. ^b^Information from Fuentes-Cardenas et al. (2022).

**Table 4:** Genome size (2C DNA) of accessions from the Peruvian Andean maize race *Cabanita* in function of their ISSR genetic structure.

Groups	2C DNA (pg)	CV^a^ (%)
G1.a	5.52 ± 0.02	0.39
G1.b	5.33 ± 0.04	0.82
G1.c	5.40 ± 0.08	1.43
G2.a	5.34 ± 0.03	0.54
G2.b	5.32 ± 0.03	0.53
G2.c	5.37 ± 0.09	1.64
G3	5.32 ± 0.07	1.25
G4	5.36 ± 0.10	1.94
G5.a	5.44 ± 0.23	4.20
G5.b	5.50 ± 0.07	1.22
G5.c	5.46 ± 0.14	2.65
G6	5.45 ± 0.10	1.75

a CV: coefficient of variation.

**Table 5:** Significant correlations between the genome size (2C DNA) of accessions from the Peruvian Andean maize race *Cabanita* and phenotypical traits.

Traits	R^2^	p value
Latitude	-0.431	0.002*
Longitude	0.322	0.026*
Bound p-coumaric acid	0.379	0.008*
Free ferulic acid derivatives	0.356	0.013*
Ear center diameter	-0.299	0.039*
Number of kernels per row	-0.439	0.002*
Ear weight	-0.319	0.027*
Kernel color (Parameter a)	0.330	0.022*

*P<0.05.

### 3.3 Relations of ISSR markers with kernel, ear, and chemical phenotypical traits

**Tables 6**–**8** shows some preliminary relations between ISSR molecular markers and kernel, ear, and phenolic compounds phenotypical traits previously measured in *Cabanita* maize accessions (Fuentes-Cardenas et al., 2022). The number of total ISSR markers per primer and per trait are also exhibited (**Tables 6**-**8**). Therefore, markers at higher frequency and that would be related to differences in phenotypical traits between genetic groups were preliminary identified (**Supplementary Tables 5**, **7**, **10**). In case of kernel, almost all evaluated traits (kernel weight, length, width, thickness, and color parameter a*) showed relation with several markers (p<0.01 and FDR<0.05). For weight, UBC810 marker was more frequent (8 markers), followed by UBC840* (4 markers), and A5 (4 markers) (**Table 6**; **Supplementary Table 5**). Kernel width was more related to PISSR14, whereas thickness with PISSR14 and UBC810 markers (**Table 6**; **Supplementary Table 5**). Kernel color (parameter a*) was related to UBC812 (3 markers) marker (**Table 6**; **Supplementary Table 5**). The genetic groups with the major contrasting results associated with kernel traits were G5.a and G1.a/G1.b. These groups also corresponded to nucleotypes with high and low 2C DNA CV values, respectively.

**Table 6:** Preliminary relations between ISSR molecular markers and kernel phenotypical traits in accessions from the Peruvian Andean maize race *Cabanita*.

Kernel trait	Groups	P value	FDR^b^	R^2^ Adj (%)	Markers^a^						
					PISSR14	UBC810	UBC817	UBC840	UBC840*	A5	UBC812
Weight	G3 vs G4	0.0076	0.0394	68	0	3	0	0	0	0	1
	G5.a vs G1.a	0.0083	0.0291	82	0	4	0	2	1	2	1
	G5.a vs G1.b	0.0021	0.0294	72	2	0	0	0	0	1	0
	G5.a vs G2.c	0.0029	0.0203	70	1	1	0	0	3	1	0
Length	G6 vs G1.b	0.0040	0.0240	62	0	1	1	0	0	0	0
Width	G2.a vs G5.a	0.0010	0.0065	83	0	0	0	0	2	1	0
	G5.a vs G1.b	0.0004	0.0052	83	2	0	0	0	0	1	0
	G5.a vs G1.c	0.0023	0.0100	72	1	0	0	1	0	1	0
	G5.b vs G5.a	0.0097	0.0315	65	1	0	0	0	0	0	0
Thickness	G3 vs G1.b	0.0031	0.0093	76	2	4	0	0	0	0	0
	G5.a vs G1.b	0.0019	0.0114	73	2	0	0	0	0	1	0
Color (Parameter a)	G1.a vs G3	0.0011	0.0044	97	1	1	0	2	1	0	3

a Number of ISSR markers per primer. ^b^ False Discovery Rate. P<0.01 and FDR<0.05 values were considered.

**Table 7:** Preliminary relations between ISSR molecular markers and ear phenotypical traits in accessions from the Peruvian Andean maize race *Cabanita*.

Ear trait	Groups	P value	FDR^b^	R^2^ Adj (%)	Markers^a^						
					PISSR14	UBC810	UBC817	UBC840	UBC840*	A5	UBC812
Length	G1.a vs G5.b	0.0020	0.0050	90	2	3	1	2	1	1	0
	G4 vs G5.b	0.0070	0.0117	84	0	0	2	0	0	1	0
	G5.b vs G3	0.0010	0.0050	90	1	3	0	0	0	0	0
Center Diameter	G2.a vs G2.b	0.0010	0.0070	88	0	0	0	0	0	0	1
	G2.a vs G5.c	0.0030	0.0105	65	0	0	0	0	0	2	0
Pith Diameter	G2.a vs G5.c	0.0010	0.0120	73	0	0	0	0	0	2	0
	G4 vs G5.c	0.0030	0.0090	76	0	0	0	0	0	1	0
	G5.c vs G1.b	0.0020	0.0080	65	1	0	1	0	0	1	0
	G5.c vs G1.c	0.0020	0.0120	63	1	0	0	0	0	2	0
Number of rows	G2.a vs G3	0.0043	0.0120	80	0	3	0	0	1	0	1
	G4 vs G6	0.0037	0.0130	81	0	0	1	0	0	0	0
	G5.a vs G2.a	0.0034	0.0159	75	0	0	0	0	2	1	0
	G6 vs G1.b	0.0017	0.0119	69	0	1	1	0	0	0	0
	G6 vs G1.c	0.0009	0.0126	74	0	0	0	1	0	0	0
Number of kernels per row	G1.a vs G5.b	0.0060	0.0216	85	2	3	1	2	1	1	0
	G4 vs G5.b	0.0016	0.0072	92	0	0	2	0	0	1	0
	G5.b vs G1.b	0.0004	0.0072	83	2	1	1	0	0	1	0
	G5.b vs G1.c	0.0065	0.0195	63	1	0	0	1	0	1	0
	G5.c vs G1.b	0.0011	0.0099	68	1	0	1	0	0	1	0
	G6 vs G1.b	0.0012	0.0072	72	0	1	1	0	0	0	0
Weight	G5.b vs G1.b	0.0008	0.0080	79	2	1	1	0	0	1	0
	G5.c vs G1.b	0.0049	0.0163	56	1	0	1	0	0	1	0
	G6 vs G1.b	0.0015	0.0075	70	0	1	1	0	0	0	0

a Number of ISSR markers per primer. ^b^ False Discovery Rate. P<0.01 and FDR<0.05 values were considered.

**Table 8:** Preliminary relations between ISSR molecular markers and phenolic compound phenotypic traits in accessions from the Peruvian Andean maize race *Cabanita*.

Phenolic compound	Groups	P value	FDR^b^	R^2^ Adj (%)	Markers^a^						
					PISSR14	UBC810	UBC817	UBC840	UBC840*	A5	UBC812
Free p-coumaric acid derivatives	G2.c vs G5.b	0.0040	0.0200	68	0	0	0	0	3	1	0
	G5.b vs G6	0.0037	0.0370	68	1	0	0	0	0	0	0
Free ferulic acid derivatives	G1.a vs G6	0.0011	0.0088	88	0	4	1	2	1	0	1
	G3 vs G6	0.0007	0.0112	85	0	3	0	0	0	0	0
Bound ferulic acid derivatives	G1.b vs G3	0.0076	0.0127	69	2	4	0	0	0	0	0
	G1.b vs G6	0.0007	0.0035	75	0	1	1	0	0	0	0
	G1.c vs G3	0.0045	0.0100	72	1	2	0	1	0	0	0
	G1.c vs G6	0.0004	0.0040	79	0	0	0	1	0	0	0
	G2.a vs G3	0.0068	0.0124	76	0	3	0	0	1	0	1
	G2.b vs G3	0.0039	0.0098	87	1	3	1	0	0	0	0
	G2.c vs G3	0.0008	0.0032	85	1	3	0	0	2	0	0
	G2.c vs G5.a	0.0062	0.0124	63	1	1	0	0	3	1	0
	G2.c vs G6	0.0001	0.0020	87	1	0	0	0	0	0	0
	G4 vs G6	0.0006	0.0040	91	0	0	1	0	0	0	0
	G5.b vs G6	0.0024	0.0080	72	1	0	0	0	0	0	0
	G5.c vs G6	0.0026	0.0074	61	0	0	1	0	0	1	0

a Number of ISSR markers per primer. ^b^ False Discovery Rate. P<0.01 and FDR<0.05 values were considered.

Regarding ear characteristics, most yield-linked traits showed potential relations with some ISSR markers. UBC810 was found at higher frequency in differences in length and number of rows (6 and 4 markers in total, respectively), whereas UBC817 and PISSR14 were linked to the number of kernels per row and ear weight (6 and 3 markers, respectively) (**Table 7**; **Supplementary Table 7**). Also, A5 had relation with pith diameter and number of kernels per row. Genetic groups G5.b and G5.c showed the major differences in length/number of kernels per row/ear weight, and pith diameter, respectively, when compared with G3/G4 and G1.a/G1.b/G1.c groups. The former groups had between medium and high 2C DNA CV values, whereas the later groups showed low and medium 2C DNA CV values.

Among contents of main bioactive compounds detected in *Cabanita* accessions (Fuentes-Cardenas et al., 2022), no relations between any ISSR markers and carotenoid compounds were found in *Cabanita* accessions (P>0.01 and FDR>0.05, **Supplementary Table 8**). Only phenolic compounds derived from hydroxycinnamic acids (HCA) showed relation with some ISSR markers (**Table 8**). Major genetic groups involving contrasting results linked to phenolic compound traits corresponded to G1.a/G1.b/G1.c, G2.a/G2.b/G2.c, G3, and G6. These groups also displayed from low to medium 2C DNA CV values. UBC840* marker was related with free p-coumaric acid derivatives (3 markers), and with the bound ferulic acid derivative compounds (6 markers) (**Table 8**; **Supplementary Table 10**). Furthermore, UBC810 marker was more frequent in differences found in the free ferulic acid derivatives (7 markers) and bound ferulic acid derivatives (17 markers in total) among genetic group comparisons (**Table 8**; **Supplementary Table 10**).

## 4 Discussions

Primers UBC812, A5, and UBC840* were the most informative and effective for the generation of clearly defined genetic clusters, despite their lower Na and Ne values (Filiz et al., 2024; Muhammad et al., 2017). The stability of He and I values among primers suggests that the variability between loci is consistent, which is a clearly defined attribute of ISSR markers (Aragón-Martínez et al., 2023; Shahabzadeh et al., 2020). On the other hand, UBC817 and P-ISSR14 primers generated limited information. However, their contribution was enough to strengthen observed genetic differences among sample populations. The genetic pattern shown in the dendrogram indicates a clear structure segregation based on the province of origin and reveals intra-racial genetic differences. In addition, the existence of subclusters unveils the presence of microstructures possibly related to variations in the agricultural management, in the genetic material selection by the farmer or by the historical context of each community (Lanes et al., 2014). The atypical grouping of 3 accessions (M23, M38, and M48) along with the AMOVA distribution are consistent with the nature of the traditional agricultural systems and the type of reproduction of this crop in the Andes (Gonhi et al., 2025). These results suggest the existence of historical and contemporary gene flow between these two provinces, maybe due to seed exchange, farmer migration, or traditional barter networks (Delêtre et al., 2011).

Based on the genetic diversity parameters associated with the genotype, a slightly higher genetic heterogeneity was revealed within the Castilla group. This characteristic may be associated with a more dynamic gene flow, better seed mixing, or more diverse agricultural management practices within this province (Balma et al., 2005; Thomas et al., 2012, and Ross-Ibarra et al., 2009). On the contrary, maize accessions from Caylloma province had higher homogeneity probably due to more rigorous selection, restrictive environmental factors, or limited germplasm flow among farmers (Coomes et al., 2015). The observation that most maize accessions from Castilla subclusters derived from different locations likely indicates that seed exchange might have been more intensive within Castilla. Other studies with Andean maize have also shown genetic differences between different geographical regions mainly influenced by the agronomic management, altitudinal gradients, and local microadaptations (Tapia et al., 2021; Rivas et al., 2022). In a previous study where current evaluated maize accessions were collected, the agricultural practices by farmers from both evaluated provinces were somewhat different (Fuentes-Cardenas et al., 2022). Differences in crop rotation and fertilization practices have been reported in both provinces (Fuentes-Cardenas et al., 2022). Other differences in relation to other plant species simultaneously grown with maize plants have been pointed out in same study (Fuentes-Cardenas et al., 2022). In addition, more extreme climatic conditions were registered in Castilla (higher temperature ranges) than in Caylloma province during *Cabanita* maize cultivation (**Supplementary Table 3**). These factors may have also played a role in observed genetic differences between these two provinces. The low PIC values shown in both provinces indicate that evaluated *Cabanita* maize accessions have low genetic variability (Hartings et al., 2008). This agrees with the AMOVA results, that confirmed the low genetic variability between maize populations from both provinces, but higher variability within each province. This is common in native maize populations, where the frequent gene flow, cross-pollination, and traditional management may lead to increased intrapopulation variability (Thomas et al., 2012). Although the variation between provinces was small, it was sufficient to show a different pattern based on the geographical origin, as was also observed in the dendrogram. Similarly, the genetic diversity of maize from Western highlands of Guatemala was higher locally more than regionally (Van Etten et al., 2008).

The 2C DNA contents in the genus *Zea* have shown wide variation, ranging from 4.20 to 11.36 pg (González et al., 2022). Realini et al. (2016) highlighted that intraspecific variation in genome size helps understand the evolutionary dynamics of DNA, mainly reflecting changes in repetitive sequences rather than in the number of genes. These variations are associated with adaptation to different environments and influence key biological processes, such as growth rate, cell cycle duration, and developmental time. They can also be related to phenotypic differences between populations of the same species (Bennett, 1971, 1972). In the case of maize races, the 2C DNA content shows notable variation, ranging from 4.20 to 6.75 pg (González et al., 2022; Realini et al., 2016), indicating high genomic heterogeneity. This variability constitutes a key element in the evolution of maize, since it helps explain the great morphological and genetic diversity that characterizes local races, resulting from historical processes of differentiation, adaptation, and selection (Matsuoka et al., 2002).

**Figure 2:**
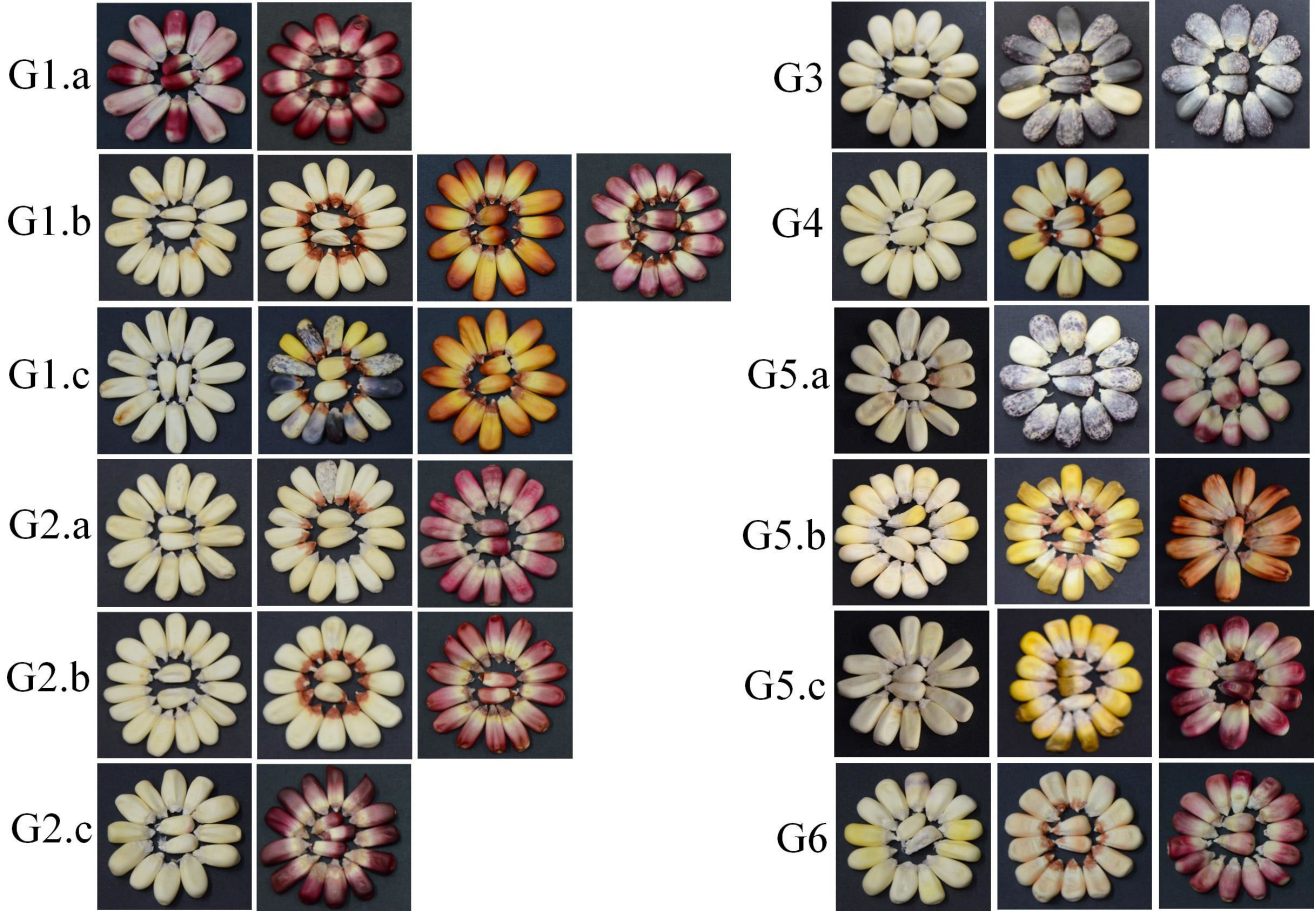
Kernel color diversity of accessions from the Peruvian Andean maize race *Cabanita* across 368 (Caylloma; groups: G1.a, G1.b, G1.c, G2.a, G2b, G2c) and 554 (Castilla; groups: G3, G4, G5.a, G5.b, G5.c, G6) meters of altitudinal gradient (kernel scaling comparisons are shown in Ranilla et al., 2023).

In current study, a limited 2C DNA intra-racial variability was observed. In addition, genetic subclusters found in Castilla province (**Figure 1**) exhibited slightly higher 2C DNA variability than maize from Caylloma. This could be related to the narrow genetic diversity observed in maize accessions from Caylloma compared to those from Castilla, as demonstrated previously by the values of Na, Ne, and He. In fact, Castilla maize samples were grown within 554 m of altitude gradient compared to Caylloma (368 m of altitude gradient). Furthermore, *Cabanita* maize grown in Caylloma province showed more homogeneous physical characteristics and higher yield, whereas that grown in Castilla showed higher phenolic content and greater antioxidant capacity (Fuentes-Cardenas et al., 2022). Together, these findings suggest a potential role of genome size in phenotypical plasticity and adaptive divergence (Šmarda and Bureš, 2010). This may be evidenced in **Figure 2**, where most genetic groups contained kernels with variable colors. However, this should be better evaluated in further studies using Genotyping-by-Sequencing (GBS).

It is most likely that the 2C DNA observed in *Cabanita* genetic groups would be shaping cell and organismal properties, likely influencing cell and nuclear size, cell cycle duration, and growth and development rates (Bennett, 1971, 1972; Cavalier-Smith, 1978, 1985; Gregory, 2005; Knight et al., 2005). In maize, the genome size variation reflects the presence of supernumerary (B) chromosomes and differences in heterochromatin (Šmarda and Bureš, 2010), which is largely composed of repetitive DNA, particularly retrotransposons, key drivers of genome evolution and seed color patterns (Šmarda and Bureš, 2010; Bilinski et al., 2018). Consistently, the genome size variation correlated with chemical, phenotypical, and geographical traits in *Cabanita* maize. These associations, particularly with phenotypical and geographical variables, are consistent with previous reports (Pegington and Rees, 1970; Beaulieu et al., 2007; Gregory et al., 2000; Díez et al., 2013). This study provides the first evidence of a correlation between genome size and metabolic traits, specifically bound p-coumaric acid and free ferulic acid derivatives.

ISSR markers have been mainly applied for the analysis of the genetic diversity and genetic structure of maize from different origins and of other crop species (Hernández et al., 2025; Do Amaral Júnior et al., 2011; Çilesiz, 2025). However, some recent studies have shown associations between ISSR markers such as UBC810 and UBC840 with some phenotypical traits in different plant species. Marker UBC810 has shown high correlation with nut weight in almond (*Prunus dulcis*) (Khadivi et al., 2023). A significant relation was found between UBC840 with grain diameter and root length in a study of a wild wheat relative (*Aegilops tauschii*) under drought and non-stress conditions (Falaknaz et al., 2025). Based on these previous studies, potential relations were further explored between ISSR markers and phenotypical traits previously determined in *Cabanita* maize accessions (Fuentes-Cardenas et al., 2022). The main aspect that stands out from obtained results is that significant differences between two genetic groups (P<0.01) in relation to a specific phenotypic trait are related with the province of origin. This occurred in around 85%, 91% and 88% of the genetic group comparisons for kernel, ear, and phenolic compound trait relations, respectively. This agrees with the genetic pattern based on ISSR markers and shown in **Figure 1**, where an influence of the province of origin was also observed.

Overall, UBC810 and UBC840* markers were more frequent in differences based on kernel, ear, and phenolic compound composition traits among genetic group comparisons of *Cabanita* maize. The UBC810 marker showed relation with yield-based traits such as kernel weight, ear length and number of rows. Both markers also were dominant in differences related to the phenolic composition among genetic group comparisons, particularly in case of HCA identified as p-coumaric and ferulic acid derivatives (in both the free and dietary fiber-linked/insoluble fractions). Although the exact identification of these compounds was not performed in the previous study (Fuentes-Cardenas et al., 2022), they could be conjugated phenolic acids known as hydroxycinnamic acid amides (HCAAs) (Macoy et al., 2015). Several HCAAs mostly derived from ferulic and p-coumaric acids have been reported in maize both in its soluble and insoluble fractions (Bento-Silva et al., 2020; Burt et al., 2019). These compounds have shown to play a role in plant defense mechanisms and development (González-Rodríguez and García-Lara, 2024). Specific HCAAs classes have shown relation with anthocyanin pigmentation in maize samples, being recognized as co-pigments (Collison et al., 2015). The marker UBC840* has been highlighted as the most informative and effective for revealing genetic differences among dark-pigmented maize varieties (Valiyeva et al., 2019).

However, these marker-phenotypical trait relations found in this investigation require further validation. It has been stated that *Cabanita* maize race may extent to other Southern Andean regions in Peru (Ministry of Environment, 2018). Then, a broader maize sampling including these regions, with additional phenotypical trait measurements, and using GBS techniques for genome-wide association studies (GWAS), would give a more complete characterization of this underrepresented Andean maize race.

## 5 Conclusions

The genetic diversity of maize accessions (48), along with preliminary marker-phenotypical trait relations from a single Peruvian Andean maize race known as *Cabanita* have been determined for the first time in this study. *Cabanita* maize accessions from two provinces (Castilla and Caylloma) of the Andean region of Arequipa (Peru) showed low genetic variability based on 7 ISSR-markers (PIC values 0.130-0.140) and low 2C DNA variability (5.35-5.65 pg). However, the ISSR derived-dendrogram detected consistent intra-racial structuring associated with the geographic province of origin. The genetic diversity parameters associated with UBC812, A5, and UBC840* primers revealed their high discriminatory power; therefore, they were the most informative for evaluating genetic diversity in *Cabanita* maize. Maize samples from Castilla had slightly higher genetic diversity and 2C DNA contents than those from Caylloma. Preliminary analysis of marker-phenotypical trait relations revealed that UBC810 and UBC840* markers were more frequent in differences found in some *Cabanita* maize traits such as physical characteristics (kernel weight, ear length and number of rows) and chemical traits (free and dietary fiber-linked hydroxycinnamic acid derivative compounds) among genetic groups. However, future GWAS need to be developed considering additional maize sampling from the other Southern Andean regions where *Cabanita* race is likely extended and expanding the evaluation of further phenotypical traits. This in turn would help to continue with applied research studies targeted to determine the designation of origin of this maize, to promote local breeding strategies, and to foster the creation of racial composites to better preserve genetic diversity within Peruvian maize races for the benefit of Peruvian Southern Andean farmers.

## Data Availability

The original contributions presented in the study are included in the article/**Supplementary Material**. Further inquiries can be directed to the corresponding author.
