# Supplementary material for: Integrating genome size variation, ISSR-derived genetic structure, and phenotypical traits from the Southern Peruvian Andean maize (Zea mays L.) race Cabanita
Source: Front Plant Sci. 2026 Jun 26;17:1798629. doi: 10.3389/fpls.2026.1798629 (PMC13350064; doi:10.3389/fpls.2026.1798629)
Supplement: Supplementary file 3 [file Table3.docx]

Supplementary Tables 1 – 10

**Table 1.** Codification, province and location of origin of evaluated 48 Cabanita maize accessions (*Zea mays* L.) used in this study

| Codes used  in current study | Previous  code accessions^1^ | Origin^a^ | | |
| --- | --- | --- | --- | --- |
|  |  | Location | District | Province |
| M1 | CPW-a | Pulluguaya | Chachas | CASTILLA |
| M2 | CPW-b |  |  |  |
| M3 | CPW-c |  |  |  |
| M4 | CPM-a |  |  |  |
| M5 | CPM-b |  |  |  |
| M6 | CPM-c |  |  |  |
| M7 | CSW-a | Subna | Ayo |  |
| M8 | CSW-b |  |  |  |
| M9 | CSW-c |  |  |  |
| M10 | CSR-a |  |  |  |
| M11 | CSR-b |  |  |  |
| M12 | CSR-c |  |  |  |
| M13 | CAY-a | Ajocha | Andahua |  |
| M14 | CAY-b |  |  |  |
| M15 | CAY-c |  |  |  |
| M16 | CAY-d |  |  |  |
| M17 | CALR-a | Alleachaya | Chachas |  |
| M18 | CALR-b |  |  |  |
| M19 | CALR-c |  |  |  |
| M20 | CALR-d |  |  |  |
| M21 | CHY-a | Huancarani | Andahua |  |
| M22 | CHY-b |  |  |  |
| M23 | CHY-c |  |  |  |
| M24 | CCR-a | Cusqui | Cabanaconde | CAYLLOMA |
| M25 | CCR-b |  |  |  |
| M26 | CCR-c |  |  |  |
| M27 | CCR-d |  |  |  |
| M28 | CCY-a |  |  |  |
| M29 | CCY-b |  |  |  |
| M30 | CCY-c |  |  |  |
| M31 | CCY-d |  |  |  |
| M32 | CLY-a | Liguay |  |  |
| M33 | CLY-b |  |  |  |
| M34 | CLY-c |  |  |  |
| M35 | CLY-d |  |  |  |
| M36 | CAW-a | Auqui |  |  |
| M37 | CAW-b |  |  |  |
| M38 | CAW-c |  |  |  |
| M39 | CAR-a |  |  |  |
| M40 | CAR-b |  |  |  |
| M41 | CAR-c |  |  |  |
| M42 | COM-a | Occollina-Tuntuiguita |  |  |
| M43 | COM-b |  |  |  |
| M44 | COM-c |  |  |  |
| M45 | COM-d |  |  |  |
| M46 | CHW-a | Huancce-Tranca |  |  |
| M47 | CHW-b |  |  |  |
| M48 | CHW-c |  |  |  |

^a^Information adapted from Fuentes-Cárdenas et al. (2022)

**Table 2.** Environmental conditions during the cultivation of *Cabanita* maize plants (average values from August 2018 to May 2019)

| **Province** | **District** | **Maximum**  **Temperature (°C)** | **Minimum**  **Temperature (°C)** | **Rainfall**  **(mm/day)** | **Relative**  **Humidity**  **(%)** |
| --- | --- | --- | --- | --- | --- |
| CASTILLA | Andahua | 17.8 ± 0.8 | 4.7 ± 1.1 | 0.8 ± 1.7 | 58 ± 16 |
|  | Ayo | 28.8 ± 0.7 | 10.4 ± 2.4 | 0.2 ± 0.5 | 70 ± 10 |
|  | Chachas | 20.6 ± 0.7 | 7.6 ± 1.7 | 1.0 ± 1.7 | 55 ± 16 |
| CAYLLOMA | Cabanaconde | 19.2 ± 0.7 | 6.6 ± 1.0 | 0.7 ± 1.8 | 82 ± 7 |

Adapted from Fuentes-Cardenas et al. (2022) and from data of the National Meteorology and Hydrology Service of Peru, SENAMHI (https://www.senamhi.gob.pe/main.php?dp=arequipa&p=estaciones)

**Table 3.** Details of seven primers used in ISSR-PCR

| **ID** | **Primer sequence** | **Tannealing** | **Reference** |
| --- | --- | --- | --- |
| A5 | 5’-GAAGAAGAAGAAGAAGAA-3’ | 46°C | Valiyeva et al. (2019) |
| P-ISSR14 | 5’-CGTCACACACACACACACA-3’ | 56.2°C | Barcaccia et al. (2003) |
| UBC810 | 5’-GAGAGAGAGAGAGAGAT-3’ | 47.5°C | Muhammad et al. (2017) |
| UBC-812 UBC-817 | 5’-GAGAGAGAGAGAGAGAA-3’  5’-CACACACACACACACAA-3’ | 47.5°C  51°C | Muhammad et al. (2017) Valiyeva et al. (2019) |
| UBC-840 | 5’-GAGAGAGAGAGAGAGAAT-3’ | 47.5°C | Valiyeva et al. (2019) |
| UBC-840* | 5’-GAGAGAGAGAGAGAGAYT-3’ | 49.1°C | Valiyeva et al. (2019) |

*Modified by Valiyeva et al. (2019)

**Table 4**. Preliminary relations between ISSR molecular markers and kernel phenotypical traits in the Peruvian Andean maize race *Cabanita*

| **Kernel trait** | **Groups** | **P value** | **FDR^b^** | **R^2^ Adj (%)** | **Markers^a^** | | | | | | |
| --- | --- | --- | --- | --- | --- | --- | --- | --- | --- | --- | --- |
|  |  |  |  |  | **PISSR14** | **UBC810** | **UBC817** | **UBC840** | **UBC840*** | **A5** | **UBC812** |
| Weight | G1.a vs G3 | 0.5991 | 0.6989 | 0 | 1 | 1 | 0 | 2 | 1 | 0 | 3 |
|  | G1.a vs G4 | 0.9775 | 0.9975 | 0 | 1 | 3 | 1 | 2 | 1 | 0 | 1 |
|  | G1.b vs G1.a | 0.9178 | 0.9884 | 0 | 0 | 2 | 0 | 0 | 1 | 0 | 1 |
|  | G2.a vs G3 | 0.0194 | 0.0340 | 64 | 0 | 3 | 0 | 0 | 1 | 0 | 1 |
|  | G2.a vs G5.a | 0.4150 | 0.5281 | 0 | 0 | 0 | 0 | 0 | 2 | 1 | 0 |
|  | G3 vs G1.b | 0.0121 | 0.0339 | 61 | 2 | 4 | 0 | 0 | 0 | 0 | 0 |
|  | G3 vs G1.c | 0.0395 | 0.553 | 46 | 1 | 2 | 0 | 1 | 0 | 0 | 0 |
|  | G3 vs G2.b | 0.0125 | 0.2917 | 62 | 2 | 3 | 1 | 0 | 0 | 0 | 0 |
|  | G3 vs G2.c | 0.0125 | 0.0250 | 62 | 1 | 3 | 0 | 0 | 2 | 0 | 0 |
|  | G4 vs G5.a | 0.0197 | 0.0306 | 72 | 0 | 1 | 0 | 0 | 0 | 1 | 1 |
| Length | G1.a vs G3 | 0.1331 | 0.1331 | 44 | 1 | 1 | 0 | 2 | 1 | 0 | 3 |
|  | G1.b vs G1.a | 0.0195 | 0.0390 | 64 | 0 | 2 | 0 | 0 | 1 | 0 | 1 |
|  | G2.b vs G1.b | 0.0134 | 0.0402 | 61 | 0 | 1 | 1 | 0 | 0 | 0 | 0 |
|  | G3 vs G4 | 0.1314 | 0.1577 | 45 | 0 | 3 | 0 | 0 | 0 | 0 | 1 |
|  | G4 vs G1.b | 0.0236 | 0.0354 | 61 | 2 | 1 | 1 | 0 | 0 | 0 | 0 |
| Width | G1.a vs G3 | 0.1078 | 0.1168 | 51 | 1 | 1 | 0 | 2 | 1 | 0 | 3 |
|  | G2.a vs G3 | 0.0228 | 0.0329 | 61 | 0 | 3 | 0 | 0 | 1 | 0 | 1 |
|  | G3 vs G1.b | 0.0152 | 0.0282 | 60 | 2 | 4 | 0 | 0 | 0 | 0 | 0 |
|  | G3 vs G1.b | 0.0497 | 0.0646 | 42 | 1 | 2 | 0 | 1 | 0 | 0 | 0 |
|  | G3 vs G2.b | 0.0917 | 0.1084 | 44 | 2 | 3 | 1 | 0 | 0 | 0 | 0 |
|  | G3 vs G4 | 0.1450 | 0.1450 | 42 | 0 | 3 | 0 | 0 | 0 | 0 | 1 |
|  | G4 vs G5.a | 0.0132 | 0.0286 | 77 | 0 | 1 | 0 | 0 | 0 | 1 | 1 |
|  | G5.a vs G1.a | 0.0115 | 0.0299 | 79 | 0 | 4 | 0 | 2 | 1 | 2 | 1 |
|  | G5.a vs G2.b | 0.0156 | 0.0254 | 67 | 2 | 0 | 1 | 0 | 0 | 2 | 0 |
| Thickness | G1.a vs G3 | 0.1013 | 0.1013 | 53 | 1 | 1 | 0 | 2 | 1 | 0 | 3 |
|  | G3 vs G2.b | 0.0427 | 0.0641 | 60 | 2 | 3 | 1 | 0 | 0 | 0 | 0 |
|  | G5.a vs G1.a | 0.784 | 0.9408 | 48 | 0 | 4 | 0 | 2 | 1 | 2 | 1 |
|  | G5.a vs G2.b | 0.0348 | 0.0696 | 55 | 2 | 0 | 1 | 0 | 0 | 2 | 0 |
| Color  (parameter a) | G1.a vs G5.a | 0.0113 | 0.0226 | 79 | 1 | 4 | 0 | 2 | 1 | 2 | 1 |
|  | G1.a vs G4 | 0.1285 | 0.1285 | 64 | 1 | 3 | 0 | 2 | 1 | 0 | 1 |
|  | G1.a vs G5.c | 0.0661 | 0.0881 | 37 | 0 | 3 | 1 | 1 | 1 | 3 | 1 |
| Color  (parameter b) | G1.a vs G1.c | 0.030 | 0.0514 | 57 | 0 | 2 | 0 | 0 | 0 | 0 | 3 |
|  | G1.c vs G3 | 0.0271 | 0.0650 | 52 | 1 | 2 | 0 | 1 | 0 | 0 | 0 |
|  | G1.c vs G5.a | 0.0328 | 0.0492 | 43 | 1 | 0 | 0 | 1 | 0 | 1 | 0 |
|  | G2.c vs G5.b | 0.0280 | 0.0560 | 45 | 0 | 0 | 0 | 0 | 3 | 1 | 0 |
|  | G3 vs G5.b | 0.0130 | 0.0780 | 69 | 1 | 3 | 0 | 0 | 0 | 0 | 0 |
|  | G5.a vs G5.b | 0.0215 | 0.0645 | 55 | 1 | 0 | 0 | 0 | 0 | 0 | 0 |
|  | G5.b vs G6 | 0.0188 | 0.0752 | 51 | 1 | 0 | 0 | 0 | 0 | 0 | 0 |
|  | G1.a vs G4 | 0.0605 | 0.0726 | 82 | 1 | 3 | 0 | 2 | 1 | 0 | 1 |
|  | G2.a vs G5.b | 0.0764 | 0.0833 | 34 | 0 | 0 | 0 | 0 | 2 | 1 | 0 |
|  | G2.b vs G5.b | 0.0520 | 0.0693 | 48 | 1 | 0 | 1 | 0 | 0 | 1 | 1 |
|  | G4 vs G5.a | 0.1415 | 0.1415 | 32 | 0 | 1 | 0 | 0 | 0 | 0 | 1 |
|  | G1.a vs G5.b | 0.0089 | 0.1068 | 81 | 2 | 4 | 1 | 2 | 1 | 1 | 0 |
| Luminosity  (L) | G1.a vs G3 | 0.1923 | 0.1923 | 31 | 1 | 1 | 0 | 2 | 1 | 0 | 3 |
|  | G1.a vs G4 | 0.1181 | 0.3545 | 67 | 1 | 3 | 0 | 2 | 1 | 0 | 1 |
|  | G1.a vs G5.b | 0.1258 | 0.1258 | 35 | 2 | 4 | 1 | 2 | 1 | 1 | 0 |

^a^Number of ISSR markers by primer. ^b^ False Discovery Rate.

**Table 5.** Total number of markers per primer and kernel trait (only for relations with P<0.01 and FDR<0.05)

| **Kernel**  **Trait** | **PISSR14** | **UBC810** | **UBC817** | **UBC840** | **UBC840*** | **A5** | **UBC812** |
| --- | --- | --- | --- | --- | --- | --- | --- |
| Weight | 3 | 8^a^ | 0 | 2 | 4^a^ | 4^a^ | 2 |
| Length | 0 | 1 | 1 | 0 | 0 | 0 | 0 |
| Width | 4^a^ | 0 | 0 | 1 | 2 | 3 | 0 |
| Thickness | 4^a^ | 4^a^ | 0 | 0 | 0 | 1 | 0 |
| Color (a) | 1 | 1 | 0 | 2^a^ | 1 | 0 | 3^a^ |

^a^Number of markers with the highest presence, per trait

**Table 6**. Preliminary relations between ISSR molecular markers and ear phenotypical traits in the Peruvian Andean maize race *Cabanita*

| **Ear trait** | **Groups** | **P value** | **FDR^b^** | **R^2^ Adj (%)** | **Markers^a^** | | | | | | |
| --- | --- | --- | --- | --- | --- | --- | --- | --- | --- | --- | --- |
|  |  |  |  |  | **PISSR14** | **UBC810** | **UBC817** | **UBC840** | **UBC840^*^** | **A5** | **UBC812** |
| Length | G1.a vs G3 | 0.309 | 0.3090 | 11 | 1 | 1 | 0 | 2 | 1 | 0 | 3 |
|  | G1.a vs G4 | 0.307 | 0.3837 | 16 | 1 | 3 | 1 | 2 | 1 | 0 | 1 |
| Tip  diameter | G1.1a vs G4 | 0.14 | 0.1750 | 61 | 1 | 3 | 1 | 2 | 1 | 0 | 1 |
|  | G3 vs G4 | 0.279 | 0.2790 | 16 | 0 | 3 | 0 | 0 | 0 | 0 | 1 |
|  | G4 vs G1.c | 0.068 | 0.1700 | 42 | 1 | 0 | 0 | 1 | 0 | 0 | 0 |
|  | G4 vs G5.a | 0.053 | 0.2650 | 56 | 0 | 1 | 0 | 0 | 0 | 1 | 1 |
|  | G4 vs G5.b | 0.072 | 0.1200 | 50 | 0 | 0 | 2 | 0 | 0 | 1 | 0 |
| Center  diameter | G1.a vs G2.a | 0.039 | 0.0455 | 62 | 0 | 2 | 0 | 2 | 3 | 0 | 0 |
|  | G2.a vs G3 | 0.175 | 0.1750 | 20 | 0 | 3 | 0 | 0 | 1 | 0 | 1 |
|  | G2.a vs G4 | 0.017 | 0.0350 | 74 | 0 | 1 | 0 | 0 | 2 | 0 | 1 |
|  | G2.a vs G5.a | 0.024 | 0.0336 | 53 | 0 | 0 | 0 | 0 | 2 | 1 | 0 |
|  | G2.a vs G5.b | 0.02 | 0.0350 | 56 | 0 | 0 | 0 | 0 | 2 | 1 | 0 |
|  | G2.a vs G6 | nd | nd | nd | nd | nd | nd | nd | nd | nd | nd |
| Pith  diameter | G1.a vs G3 | 0.172 | 0.1876 | 35 | 1 | 1 | 0 | 2 | 1 | 0 | 3 |
|  | G1.a vs G5.c | 0.02 | 0.0400 | 56 | 0 | 3 | 1 | 1 | 1 | 3 | 1 |
|  | G3 vs G1.b | 0.044 | 0.0587 | 44 | 2 | 4 | 0 | 0 | 0 | 0 | 0 |
|  | G3 vs G1.c | 0.055 | 0.0660 | 40 | 1 | 2 | 0 | 1 | 0 | 0 | 0 |
|  | G3 vs G2.a | 0.02 | 0.0343 | 63 | 0 | 3 | 0 | 0 | 0 | 0 | 1 |
|  | G3 vs G4 | 0.021 | 0.0315 | 83 | 0 | 3 | 0 | 0 | 0 | 0 | 1 |
|  | G4 vs G5.a | 0.011 | 0.0264 | 79 | 0 | 1 | 0 | 0 | 0 | 1 | 1 |
|  | G4 vs G5.c | 0.308 | 0.3080 | 2 | 0 | 0 | 1 | 0 | 0 | 1 | 0 |
| Rachis  diameter | G1.a vs G2.b | 0.120 | 0.1543 | 48 | 0 | 4 | 1 | 2 | 0 | 0 | 3 |
|  | G1.a vs G3 | 0.193 | 0.2171 | 31 | 1 | 1 | 0 | 2 | 1 | 0 | 3 |
|  | G1.a vs G5.c | 0.039 | 0.1755 | 46 | 0 | 3 | 1 | 1 | 1 | 3 | 1 |
|  | G1.a vs G6 | 0.039 | 0.3510 | 53 | 0 | 2 | 1 | 1 | 1 | 0 | 0 |
|  | G3 vs G4 | 0.252 | 0.2520 | 20 | 0 | 3 | 0 | 0 | 0 | 0 | 1 |
|  | G4 vs G2.b | 0.108 | 0.1620 | 51 | 2 | 1 | 2 | 0 | 0 | 0 | 0 |
|  | G4 vs G5.a | 0.054 | 0.1215 | 56 | 0 | 1 | 0 | 0 | 0 | 1 | 1 |
|  | G4 vs G5.c | 0.074 | 0.1332 | 35 | 0 | 0 | 0 | 0 | 0 | 1 | 0 |
|  | G4 vs G6 | 0.049 | 0.1470 | 49 | 0 | 0 | 1 | 0 | 0 | 0 | 0 |
| Corncob  diameter | G1.a vs G3 | 0.1756 | 0.1756 | 35 | 1 | 1 | 0 | 2 | 1 | 0 | 3 |
|  | G5.a vs G1.a | 0.0606 | 0.0758 | 53 | 0 | 4 | 0 | 2 | 1 | 2 | 1 |
|  | G5.a vs G1.b | 0.037 | 0.0925 | 41 | 2 | 0 | 0 | 0 | 0 | 1 | 0 |
|  | G5.a vs G2.a | 0.0139 | 0.0695 | 61 | 0 | 0 | 0 | 0 | 2 | 1 | 0 |
|  | G5.a vs G4 | 0.0577 | 0.0962 | 54 | 0 | 1 | 0 | 0 | 0 | 1 | 1 |
| Number  of rows | G1.a vs G2.a | 0.13 | 0.1655 | 34 | 0 | 2 | 0 | 2 | 3 | 0 | 0 |
|  | G1.a vs G6 | 0.2067 | 0.2226 | 16 | 0 | 2 | 1 | 1 | 1 | 0 | 0 |
|  | G2.a vs G4 | 0.0105 | 0.0245 | 80 | 0 | 1 | 0 | 0 | 2 | 0 | 1 |
|  | G2.a vs G6 | nd | nd | nd | nd | nd | nd | nd | nd | nd | nd |
|  | G3 vs G1.b | 0.0276 | 0.0429 | 51 | 2 | 4 | 0 | 0 | 0 | 0 | 0 |
|  | G3 vs G1.c | 0.0251 | 0.0439 | 53 | 1 | 2 | 0 | 1 | 0 | 0 | 0 |
|  | G3 vs G2.b | 0.0582 | 0.0815 | 54 | 2 | 3 | 1 | 0 | 0 | 0 | 0 |
|  | G3 vs G4 | 0.1678 | 0.1958 | 36 | 0 | 3 | 0 | 0 | 0 | 0 | 1 |
|  | G4 vs G1.c | 0.3164 | 0.3164 | 4 | 1 | 0 | 0 | 1 | 0 | 0 | 0 |
|  | G5.a vs G1.c | 0.0110 | 0.0220 | 57 | 1 | 0 | 0 | 1 | 0 | 1 | 0 |
| Number of kernels  per row | G1.a vs G4 | 0.0382 | 0.0458 | 89 | 1 | 3 | 1 | 2 | 1 | 0 | 1 |
|  | G1.a vs G5.c | 0.0332 | 0.0427 | 48 | 0 | 3 | 1 | 1 | 1 | 3 | 1 |
|  | G1.a vs G6 | 0.0107 | 0.0241 | 71 | 0 | 2 | 1 | 1 | 1 | 0 | 0 |
|  | G4 vs G1.c | 0.1785 | 0.1785 | 19 | 1 | 0 | 0 | 1 | 0 | 0 | 0 |
|  | G4 vs G2.c | 0.0536 | 0.0603 | 47 | 0 | 0 | 0 | 0 | 3 | 0 | 1 |
|  | G4 vs G5.a | 0.1534 | 0.1624 | 30 | 0 | 1 | 0 | 0 | 0 | 1 | 1 |
|  | G5.b vs G2.a | 0.0179 | 0.0322 | 57 | 0 | 0 | 0 | 0 | 2 | 1 | 0 |
|  | G5.b vs G2.b | 0.0224 | 0.0336 | 62 | 1 | 0 | 1 | 0 | 0 | 1 | 1 |
|  | G5.b vs G2.c | 0.0182 | 0.0298 | 51 | 0 | 0 | 0 | 0 | 3 | 1 | 0 |
|  | G5.b vs G3 | 0.0104 | 0.0267 | 71 | 1 | 3 | 0 | 0 | 0 | 0 | 0 |
|  | G5.c vs G3 | 0.0331 | 0.0458 | 43 | 0 | 3 | 0 | 0 | 0 | 0 | 0 |
|  | G6 vs G5.b | 0.0157 | 0.0314 | 53 | nd | nd | nd | nd | nd | nd | nd |
| Weight | G1.a vs G5.b | 0.0715 | 0.0794 | 50 | 2 | 3 | 1 | 2 | 1 | 1 | 0 |
|  | G2.a vs G1.b | 0.01 | 0.0250 | 58 | 0 | 0 | 0 | 0 | 2 | 0 | 1 |
|  | G2.a vs G4 | 0.1593 | 0.1593 | 28 | 0 | 1 | 0 | 0 | 2 | 0 | 1 |
|  | G4 vs G5.b | 0.0381 | 0.0544 | 62 | 0 | 0 | 2 | 0 | 0 | 1 | 0 |
|  | G5.b vs G3 | 0.0144 | 0.0288 | 68 | 1 | 3 | 0 | 0 | 0 | 0 | 0 |
|  | G5.c vs G3 | 0.0493 | 0.0616 | 37 | 0 | 3 | 0 | 0 | 0 | 0 | 0 |
|  | G6 vs G3 | 0.0250 | 0.0417 | 53 | 0 | 3 | 0 | 0 | 0 | 0 | 0 |

^a^Number of ISSR markers associated by primer. ^b^ False Discovery Rate. Nd: no determined

**Table 7.** Total number of markers per primer and ear trait (only for relations with P<0.01 and FDR<0.05)

| **Ear**  **Trait** | **PISSR14** | **UBC810** | **UBC817** | **UBC840** | **UBC840*** | **A5** | **UBC812** |
| --- | --- | --- | --- | --- | --- | --- | --- |
| Length | 3 | 6^a^ | 3 | 2 | 1 | 2 | 0 |
| Center diameter | 0 | 0 | 0 | 0 | 0 | 2^a^ | 1 |
| Pith diameter | 2 | 0 | 1 | 0 | 0 | 6^a^ | 0 |
| N° of rows | 0 | 4^a^ | 2 | 1 | 3 | 1 | 1 |
| N° kernels per row | 6^a^ | 5^*^ | 6^a^ | 3 | 1 | 5^a^ | 0 |
| Weight | 3^a^ | 2 | 3^a^ | 0 | 0 | 2 | 0 |

^a^Number of markers with the highest presence, per trait

**Table 8**. Preliminary relations between ISSR molecular markers and carotenoid compounds phenotypical traits in the Peruvian Andean maize race *Cabanita*

| **Phenotypic characters** | **Groups** | **P value** | **FDR^b^** | **R^2^ Adj (%)** | **Markers^a^** | | | | | | |
| --- | --- | --- | --- | --- | --- | --- | --- | --- | --- | --- | --- |
|  |  |  |  |  | **PISSR14** | **UBC810** | **UBC817** | **UBC840** | **UBC840*** | **A5** | **UBC812** |
| Lutein | G4 vs G5.b | 0.1696 | 0.1696 | 26 | 0 | 0 | 2 | 0 | 0 | 1 | 0 |
|  | G5.b vs G3 | 0.0324 | 0.0648 | 56 | 1 | 3 | 0 | 0 | 0 | 0 | 0 |
| Lutein  isomers | G4 vs G1.c | 0.1031 | 0.1031 | 33 | 1 | 0 | 0 | 1 | 0 | 0 | 0 |
|  | G4 vs G5.b | 0.0899 | 0.1199 | 44 | 0 | 0 | 2 | 0 | 0 | 1 | 0 |
|  | G5.b vs G2.c | 0.0126 | 0.0504 | 56 | 0 | 0 | 0 | 0 | 3 | 1 | 0 |
|  | G5.b vs G3 | 0.0571 | 0.1142 | 46 | 1 | 3 | 0 | 0 | 0 | 0 | 0 |
| Zeaxanthin | G5.b vs G3 | 0.2059 | 0.4118 | 16 | 1 | 3 | 0 | 0 | 0 | 0 | 0 |
|  | G5.b vs G5.a | >0.999 | 0.9999 | 0 | 1 | 0 | 0 | 0 | 0 | 0 | 0 |
| Total carotenoids | G2.a vs G5.b | 0.0357 | 0.0714 | 47 | 0 | 0 | 0 | 0 | 2 | 1 | 0 |
|  | G5.b vs G2.b | 0.0861 | 0.0861 | 37 | 1 | 0 | 1 | 0 | 0 | 1 | 1 |
|  | G5.b vs G2.c | 0.0137 | 0.0548 | 55 | 0 | 0 | 0 | 0 | 3 | 1 | 0 |
|  | G5.b vs G5.a | 0.0370 | 0.0493 | 47 | 1 | 0 | 0 | 0 | 0 | 0 | 0 |

^a^Number of ISSR markers by primer. ^b^ False Discovery Rate.

**Table 9**. Preliminary relations between ISSR molecular markers and phenolic compounds phenotypical traits in the Peruvian Andean maize race *Cabanita*

| **Phenolic compound** | **Groups** | **P value** | **FDR^b^** | **R^2^ Adj (%)** | **Markers^a^** | | | | | | |
| --- | --- | --- | --- | --- | --- | --- | --- | --- | --- | --- | --- |
|  |  |  |  |  | **PISSR14** | **UBC810** | **UBC817** | **UBC840** | **UBC840*** | **A5** | **UBC812** |
| Anthocyanins | G1.a vs G1.c | 0.0293 | 0.1172 | 58 | 0 | 2 | 0 | 0 | 1 | 0 | 3 |
|  | G1.a vs G2.b | 0.1588 | 0.2117 | 38 | 0 | 5 | 1 | 2 | 0 | 0 | 3 |
|  | G1.a vs G4 | 0.2476 | 0.2476 | 35 | 1 | 3 | 1 | 2 | 1 | 0 | 0 |
|  | G1.a vs G5.b | 0.0533 | 0.1066 | 56 | 2 | 4 | 1 | 2 | 1 | 1 | 0 |
| Free p-coumaric acid | G1.a vs G3 | 0.0274 | 0.2192 | 79 | 0 | 1 | 0 | 2 | 1 | 0 | 3 |
|  | G2.a vs G5.b | 0.0352 | 0.1408 | 48 | 0 | 0 | 0 | 0 | 2 | 1 | 0 |
|  | G2.c vs G5.b | 0.0296 | 0.1578 | 45 | 0 | 0 | 0 | 0 | 3 | 1 | 0 |
|  | G5.b vs G6 | 0.0176 | 0.2816 | 52 | 1 | 0 | 0 | 0 | 0 | 0 | 0 |
|  | G1.a vs G1.b | 0.1465 | 0.1674 | 25 | 0 | 3 | 0 | 0 | 1 | 0 | 1 |
|  | G1.a vs G1.c | 0.0627 | 0.1433 | 44 | 0 | 2 | 0 | 0 | 1 | 0 | 3 |
|  | G1.a vs G2.b | 0.1170 | 0.1440 | 48 | 0 | 5 | 1 | 2 | 0 | 0 | 3 |
|  | G1.a vs G2.c | 0.0705 | 0.1253 | 41 | 1 | 2 | 0 | 1 | 4 | 0 | 3 |
|  | G1.a vs G5.a | 0.4066 | 0.4066 | 00 | 1 | 4 | 0 | 2 | 1 | 1 | 1 |
|  | G1.a vs G5.b | 0.0942 | 0.1256 | 43 | 2 | 4 | 1 | 2 | 1 | 0 | 0 |
|  | G1.a vs G5.c | 0.0888 | 0.1291 | 31 | 0 | 3 | 1 | 1 | 1 | 3 | 0 |
|  | G1.a vs G6 | 0.0722 | 0.1155 | 41 | 0 | 4 | 1 | 2 | 1 | 0 | 1 |
|  | G2.b vs G5.b | 0.0652 | 0.1304 | 43 | 2 | 0 | 1 | 0 | 0 | 1 | 1 |
|  | G3 vs G5.b | 0.0537 | 0.1718 | 47 | 1 | 3 | 0 | 0 | 0 | 0 | 0 |
|  | G3 vs G5.c | 0.0598 | 0.1594 | 34 | 0 | 3 | 0 | 0 | 0 | 0 | 0 |
|  | G4 vs G5.b | 0.1738 | 0.1853 | 26 | 0 | 0 | 2 | 0 | 0 | 1 | 0 |
| Bound p-coumaric acid | G3 vs G5.b | 0.0326 | 0.1304 | 56 | 1 | 3 | 0 | 0 | 0 | 0 | 0 |
|  | G1.a vs G5.b | 0.0843 | 0.1124 | 46 | 2 | 4 | 1 | 2 | 1 | 0 | 0 |
|  | G2.b vs G4 | 0.2998 | 0.2998 | 12 | 2 | 1 | 2 | 0 | 0 | 0 | 1 |
|  | G4 vs G5.b | 0.0624 | 0.1248 | 53 | 0 | 0 | 2 | 0 | 0 | 1 | 0 |
| Free p-coumaric acid derivatives | G1.a vs G5.b | 0.0201 | 0.0402 | 72 | 1 | 4 | 1 | 2 | 1 | 0 | 0 |
|  | G2.b vs G5.b | 0.0173 | 0.0.432 | 65 | 2 | 0 | 1 | 0 | 0 | 1 | 1 |
|  | G3 vs G5.b | 0.0134 | 0.0446 | 68 | 1 | 3 | 0 | 0 | 0 | 0 | 0 |
|  | G3 vs G5.c | 0.0340 | 0.0566 | 42 | 0 | 3 | 0 | 0 | 0 | 0 | 0 |
|  | G1.a vs G1.c | 0.0958 | 0.1197 | 35 | 0 | 2 | 0 | 0 | 1 | 0 | 3 |
|  | G1.a vs G2.a | 0.2424 | 0.2693 | 15 | 0 | 4 | 0 | 2 | 3 | 0 | 0 |
|  | G1.a vs G5.a | 0.3079 | 0.3079 | 07 | 1 | 4 | 0 | 2 | 1 | 1 | 1 |
|  | G1.a vs G5.c | 0.0541 | 0.0772 | 40 | 0 | 3 | 1 | 1 | 1 | 3 | 0 |
| Free ferulic acid | G1.a vs G5.c | 0.0648 | 0.0648 | 37 | 0 | 3 | 1 | 1 | 1 | 3 | 0 |
| Free ferulic acid derivatives | G1.a vs G2.a | 0.0475 | 0.0691 | 58 | 0 | 4 | 0 | 2 | 3 | 0 | 0 |
|  | G1.a vs G5.c | 0.0423 | 0.0677 | 44 | 0 | 3 | 1 | 1 | 1 | 3 | 0 |
|  | G2.a vs G5.b | 0.0188 | 0.0752 | 57 | 0 | 0 | 0 | 0 | 2 | 1 | 0 |
|  | G2.b vs G5.b | 0.0230 | 0.0736 | 61 | 1 | 0 | 1 | 0 | 0 | 1 | 1 |
|  | G2.b vs G5.c | 0.0310 | 0.0620 | 44 | 1 | 0 | 2 | 0 | 0 | 1 | 1 |
|  | G2.c vs G5.b | 0.0111 | 0.0592 | 57 | 0 | 0 | 0 | 0 | 3 | 1 | 0 |
|  | G3 vs G5.b | 0.0249 | 0.0664 | 60 | 1 | 3 | 0 | 0 | 0 | 0 | 0 |
|  | G3 vs G5.c | 0.0376 | 0.0668 | 41 | 0 | 3 | 0 | 0 | 0 | 0 | 0 |
|  | G1.a vs G1.b | 0.0774 | 0.0953 | 40 | 0 | 3 | 0 | 0 | 1 | 0 | 1 |
|  | G1.a vs G1.c | 0.1886 | 0.1886 | 18 | 0 | 2 | 0 | 0 | 1 | 0 | 3 |
|  | G1.a vs G3 | 0.1517 | 0.1618 | 40 | 1 | 1 | 0 | 2 | 1 | 0 | 3 |
|  | G1.a vs G5.b | 0.0501 | 0.0668 | 57 | 2 | 4 | 1 | 2 | 1 | 0 | 0 |
|  | G4 vs G5.b | 0.0829 | 0.0947 | 46 | 0 | 0 | 2 | 0 | 0 | 1 | 0 |
|  | G4 vs G6 | 0.0282 | 0.0645 | 58 | 0 | 0 | 1 | 0 | 0 | 0 | 0 |
| Bound ferulic acid derivatives | G1.a vs G6 | 0.0129 | 0.0198 | 69 | 0 | 4 | 1 | 2 | 1 | 0 | 1 |
|  | G1.b vs G5.a | 0.0155 | 0.0207 | 53 | 1 | 0 | 0 | 0 | 0 | 1 | 0 |
|  | G2.a vs G5.a | 0.0145 | 0.0207 | 60 | 0 | 0 | 0 | 0 | 2 | 1 | 0 |
|  | G2.b vs G5.a | 0.0296 | 0.0329 | 57 | 1 | 0 | 1 | 0 | 0 | 2 | 0 |
|  | G3 vs G5.b | 0.0165 | 0.0206 | 66 | 1 | 3 | 0 | 0 | 0 | 0 | 0 |
|  | G3 vs G5.c | 0.0181 | 0.0213 | 57 | 0 | 3 | 0 | 0 | 0 | 0 | 0 |
|  | G4 vs G5.a | 0.0381 | 0.0401 | 62 | 0 | 1 | 0 | 0 | 0 | 1 | 1 |
|  | G1.a vs G3 | 0.0716 | 0.0716 | 62 | 1 | 1 | 0 | 2 | 1 | 0 | 3 |

^a^Number of ISSR markers by primer. ^b^ False Discovery Rate.

**Table 10.** Total number of markers per primer and phenolic compound traits (only for relations with P<0.01 and FDR<0.05)

| **Phenolic**  **compounds** | **PISSR14** | **UBC810** | **UBC817** | **UBC840** | **UBC840*** | **A5** | **UBC812** |
| --- | --- | --- | --- | --- | --- | --- | --- |
| Free p-coumaric acid derivatives | 1 | 0 | 0 | 0 | 3^a^ | 1 | 0 |
| Free ferulic acid derivatives | 0 | 7^a^ | 1 | 2 | 1 | 0 | 1 |
| Bound ferulic acid derivatives | 8^a^ | 17^a^ | 4 | 2 | 6^a^ | 2 | 1 |

^a^Number of markers with the highest presence, per trait
